# Care, co-survival, and decency: expanding the understanding of outdoor life and smaller alcohol and drug scenes in Denmark and Norway

**DOI:** 10.3389/fpsyt.2024.1233701

**Published:** 2024-08-15

**Authors:** Jonas Strandholdt Bach, Trond Erik Grønnestad, Anne Schanche Selbekk, Vilde Holan Bye, Amanda Skjong

**Affiliations:** ^1^ Center for Alcohol and Drug Research, BSS, Aarhus University, Aarhus, Denmark; ^2^ Department of Public Health, Faculty of Health, University of Stavanger, Stavanger, Norway; ^3^ Center for Alcohol and Drug Research (KORFOR), Stavanger University Hospital, Stavanger, Norway

**Keywords:** marginalization, open drug scenes, alcohol users, drug users, public space, comparative case study, ethnography

## Abstract

**Introduction:**

Public spaces where alcohol and other drugs are openly used and marginal citizens gather, exist in many Nordic cities. The biggest open drug scenes in the Nordic countries are in cities like Oslo and Copenhagen; however, there are smaller scenes in other cities and suburbs, centered around shed-like structures, offering some form of shelter and a designated space for marginalized people involved in heavy drug and alcohol use who hang out in public space. In this paper, we investigate, in a comparative perspective, the characteristics and functions of smaller open alcohol and drug scenes, and how their existence is negotiated in the local community and among the citizens using them.

**Methods and material:**

This article is a comparative case-study based on data from fieldwork (participating observation and interviews) carried out in two specific, yet somewhat similar, locations in Denmark and Norway between 2017 – 2022. A cross-case analysis was performed to identify commonalities and differences.

**Results:**

Smaller open alcohol and drug scenes are non-regulated spaces of ambivalence and ongoing negotiation in local communities. Based on the data across locations, they represent possibilities for informal care and community for citizens in marginalized situations. The scenes are further, across location, characterized by a mutual agreement of performing decency, e.g., not allowing minor drug sale/use.

**Discussion:**

To enable public spaces as smaller alcohol and drug scenes can play a role in reducing harm for marginalized citizens. Communication and dialogue between citizens using an open drug scene and the wider community may help reduce stigma.

## 1 Introduction

Social practices regarding alcohol and other drug use are closely connected to the urban life of a city (1). These social practices take different forms, from being closely connected to nightlife in the city with its restaurants, bars, and clubs, to the more informal drinking and drug using practices of the city. Some of these urban spaces are characterized by being places where marginalized citizens gather, and as open “drug scenes”, i.e., situations where citizens are publicly confronted with drug use and drug dealing (2–4).

Terms like “open drug scene” can provide an intuitive meaning that captures an urban phenomenon, but as Bless et al. (2) argue, it also covers potentially quite divergent realities. According to Bless et al. (2), open drug scenes vary when it comes to how visible they are, how big they are, and where in the city they are sited. They can also vary according to characteristics of the people attending the scene (e.g., age), how deeply they are involved with different kinds of crime and violence, and how long they have existed (5), and might have transformed over time.

Open alcohol and drug scenes also vary (on a continuum) when it comes to one of the main characteristics of such sites; namely, how much open drug use and drug selling takes place, and how this can change over time (6, p. 39). Some (smaller) scenes where marginalized citizens gather are characterized less, or not at all, by drug dealing and drug intake, and are gathering places where alcohol is consumed and an occasional joint is shared (6–9). This directs attention not only to public nuisances, policy strategies, and crime management, but to the role these scenes play in the lives of people managing troublesome situations, spending time outdoors in urban environments (7, 10–12). The aspect of what characterizes the social practices related to the scenes, and how they represent meaning and serve purposes for the people involved, are less discussed in the existing literature. The phenomenon of open alcohol and drug scences is thus also closely related to issues of homelessness and the exclusion of marginalized groups in urban environments (6), thereby connecting them to a wider theme of social inclusion, service provision to marginalized citizens, and their rights to public space. To our knowledge there is no overall assessment of the existence of such smaller alcohol and drug scenes in either Norway or Denmark.

How a phenomenon like open drug and alcohol scenes should be considered and how it is debated is closely related to drug and alcohol policy. In a European context, there has typically been conflict between liberal versus restrictive policies, focusing respectively on addiction as a health problem and drug use behavior as a public nuisance problem (3). The challenge has been to balance the concerns of the public on one hand, and concern for persons struggling with addiction and harmful use on the other. There have been changes in national drug policies in many countries worldwide in the past decades, from a policy of zero-tolerance to decriminalization or legalization strategies (13, 14).

Although alcohol and drug policy in the Nordic countries in general has been characterized as strict, there have traditionally been differences between the countries, with Denmark considered as the most liberal (15). In the Norwegian context, the movement has been from a restrictive policy of drugs towards a policy of potential decriminalization (16), although the political debate is ongoing (17). Still, in Denmark there are tendencies towards moving from a traditional pragmatic liberalistic drug policy to a more repressive drug policy (18). When it comes to alcohol policy, Denmark has the highest recorded consumption of alcohol in the Nordic countries, and the highest proportion of the population drinking at a higher frequency (19). Denmark has traditionally, compared to the other Nordic countries, had a more liberal retail sales system, lower legal age limit for alcohol sales, and higher levels of drunk driving. Alcohol-related harm has never gained the kind of public concern here as it has in other Nordic countries (19, p. 440-441). In general, alcohol is a substance that is legal to consume in some places like bars and restaurants, but illegal in others such as parks, malls, parking lots, and so on, while other drugs are illegal altogether (1). Still, the threshold of tolerance when it comes to these urban practices is likely to be connected to more general alcohol policy and would traditionally be more restrictive in Norway compared to Denmark.

In regard to how open drug scenes are dealt with, in Norway a change has been documented from strictly punitive strategies to a mixture of harm-reducing and punitive efforts (20). Similarly in Denmark, when it comes to balancing the concern of the persons involved in the open drug scene and the public, initiatives have been made to offer coordinated help from health and social services on one hand, and police action on the other, related to open drug scenes, resulting in better knowledge among police and alternative responses other than penalty and banishment (21). In Norway and Denmark, these debates have typically been connected to drug scenes in bigger cities (20–23).

In this article, we have a twofold focus. First, we want to look closer into smaller open alcohol and drug scenes which may share some characteristics of open drug scenes in bigger cities, but that might also expand our understanding of what an open alcohol and drug scene is, containing other characteristics that are less theorized in the existing literature. Secondly, we want to take a closer look at the scenes from the “inside” – from the perspective of the people attending them, to get a better understanding of the social interactions taking place and what purposes they fill for the people involved.

Here we will explore and compare the social space of two such scenes, respectively from one city in Denmark and one in Norway. The two are sited respectively in a center of a suburb and in the center of a smaller city, offering some form of shelter and a designated space for marginalized groups of drug and alcohol users who hang out in public space. We investigate the characteristics, functions, and meanings of smaller open alcohol and drug scenes, and how their existence is negotiated in the local community, with and among the citizens using them. The aim is to gain an in-depth understanding of the phenomenon of ‘smaller’ open alcohol and drug scenes as public and social spaces.

## 2 Materials and methods

The study is designed as a comparative case study (24). There are several different approaches to case studies (25, 26). The comparative case study approach is described as a heuristic, iterative, and emergent research design, based on an interpretivist epistemology (24). As a tool to examine processes of sense-making as they develop over time and are contested in distinct settings, in relation to systems of power and inequality (24, p. 10). The approach focuses respectively on the vertical (with-in case), horizontal (cross-case), and transversal (across time) dimensions in the comparison of cases in the analysis (24).

The study is based on data from multitemporal ethnographic fieldwork (27) conducted in Norway and Denmark, in two specific but similar locations in the period from 2012 – 2022. The fieldwork in Norway consisted of weekly participant observation by one of the researchers, from June 2012 until June 2013, interacting with 70-80 persons who were part of the open alcohol and drug use scene. The period of data collection was extended with 11 qualitative interviews with eight participants and step-in-step-out fieldwork at the scene in the period from June 2013 until April 2015 (28). By step-in-step-out fieldwork we mean that the researchers did not follow the scene or the people around the clock every day, and were not as such fully immersed, but came by regularly and stepped in and stepped out of the context (29, p. 78). The researcher introduced himself to everyone and explained the reason for his presence. The first period of fieldwork was extended with additional fieldwork and qualitative interviews in the period from 2020 until 2022. Twenty-five interviews were conducted with persons who were part of the scene, and 23 interviews with representatives from social and health services, local police officers, politicians, townhall employees and city priest. The extended study was conducted by several researchers. The fieldwork in Denmark consisted of a three-year step-in-step-out fieldwork in the period from September 2019, including 10 qualitative interviews conducted in June, July, and October 2020 (8). In the period the ethnographer visited the scene regularly, on average once a week, while the duration of visits could vary from 1 to 5 hours of participant observation, consisting of sitting in the Shed and listening to and talking with the people present. In addition, the data consisted of an interview with a local community worker. Participants have been given pseudonyms in the running text.

For both the Norwegian and the Danish case, the researchers were open about the reasons for their presence and while it was to some extent possible to blend in, and take part in conversations, sharing experiences, et cetera, the people present possibly adjusted their behavior to the presence of an “outsider”. As one of the Shed-goers told the ethnographer in the Danish case; “You do know they [the others in the Shed] are on their best behavior when you’re there, right?”. While the people present might to some extent have behaved differently than if no researcher had been present, with time and the development in trust (based on not reporting to police or security guards, keeping shared information confidential, et cetera) behaviors could occasionally become rowdier, sharing and selling of drugs more open, and stories about conflict and crime shared more freely.

The analysis was based on the following questions.

- How was the scene established?
- Who attends the scene?
- What happens in the scenes and what functions does it fill for the people using it?
- What kind of contact, dialogue, and negotiations have taken place with local community and authorities?

The following steps were executed in the analysis process, based on the analytical scheme:

1. with-in case analysis of data from each country
2. comparative cross-case analysis
3. narrative synthesis

The analysis included horizontal, vertical, and transversal elements (24). The first step can be characterized as a re-analysis of existing coding from the two initial fieldworks, but where all existing text was re-coded due to the analytical scheme. In the second step, the results from the two analyses in step one was compared to each other, looking for commonalities and differences in a cross-case analysis, making codes based on the analytical questions. In the third step, central insights were synthesized and discussed in relation to terms and perspectives from the research literature. The first step was conducted by JSB and TEG from each country, based on their previous fieldwork. In Norway, supplementary data was collected by VHB and AS (master’s students) and ASS (researcher in sub-study). The second and third step was conducted by Authors JSB, TEG, and ASS.

In the analysis, the different dimension and themes were also explored in relation to different theoretical concepts, described chronologically as they appear in the text. All names of people used in the running text are pseudonyms. The fieldwork in Norway was recommended by SIKT (former NSD) for privacy consideration. The Danish research followed the ethical guidelines of Aarhus University. Informed consent has been collected from all interview participants. 3.1 og 3.2 in the findings section represent the with-in case analysis. 3.3 in the analysis represent the comparative cross-case analysis.

## 3 Findings

### 3.1 The Shed

#### 3.1.1 From informal to more formalized scene

The Danish scene, here referred to as “The Shed”, is situated in a suburb of a large Danish provincial city. It was established in its current form in the late 2000s, where the shed/shelter building was constructed at the back of a local supermarket and on the edge of the local square. Today, the shed-like structure is made of corrugated iron and measures approximately 6 by 3 meters. Inside, there are solid but worn tables and chairs. In the immediate vicinity of the Shed are a church, a supermarket, a kiosk, a library, a community center, a pizza restaurant, a mosque (in a former store), kindergartens, and a large number of social housing units.

Before the existence of the current Shed, there was a more informal drug and alcohol scene where people would hang out in front of the entrance to the local supermarket or on the stairs leading to the church entrance, or other spots at the local square. A local community worker, representing the housing organizations, met with representatives from the church, the supermarket, and other smaller local businesses to discuss the impact of the group of drug and alcohol users’ presence at the local square. They agreed that the scene was causing insecurity among other people using the communal facilities (shopping, library, church, et cetera), due to noise, fights, dogs without leashes, litter, and intoxicated, volatile behavior, and the presence of both hard drugs and large quantities of alcohol. They also agreed that it would be impossible to dissolve the scene, as many of the people in the scene lived nearby.

Initially, the group decided on building a shed-like structure to give the scene a place where people could anchor, and at the same time shielding the group of drug and alcohol users from passersby, but also the other way around, as the local community worker (who had worked in the neighborhood for 25 years) explained in an interview. A shed was then erected in a nearby park area, but the scene did not move there. It remained where it had been, in front of the church and the supermarket, and right where people who were grocery shopping, going to church or to the library, or going to pick up their kids from one of the nearby kindergartens, would pass daily.

The group which initiated the construction of the shed in the park then invited the people taking part in the drug scene to a meeting at a local bar where, as the community worker put it, “the sodas were way over the last use-by date”. When the question was raised as to why the scene did not move to the shed in the park, the response was that it was “too far away from the beers” and that they wanted to be where there were other people and some street life. The shed was then moved to a more central place, almost identical to the current placement immediately behind the supermarket and 25 meters from the square but facing a pedestrian path and the adjacent parking lot.

In the early years of The Shed, there were conflicts, particularly involving people from the drug and drinking scene and groups of local youths with predominantly immigrant backgrounds. One New Year’s Eve, The Shed was blown to pieces by powerful fireworks and, in the same time period, an unknown perpetrator stole a construction machine and used it as a battering ram to level The Shed. In both instances, The Shed was reconstructed. In recent years, on account of the community worker, the situation has been quieter and more peaceful and, while there still may be personal confrontations and conflicts involving people from the drug and alcohol scene and others from the neighborhood, the presence of The Shed and the scene is generally accepted. The kids from kindergarten wave when they pass, and dog walkers stop to chat, as do walking groups of older women organized by the church and other older people from the neighborhood.

#### 3.1.2 “The outdoor people”

The people attending the scene are sometimes referred to by the community workers and other locals as “the outdoor people”, as they spend a lot of time outside in all types of weather. Presently, the scene is attended by a mixed group of people. Over the past 15 years, it has changed from being a mixed drug and alcohol scene towards predominantly an open alcohol scene, though cannabis is sometimes used, and there are people attending The Shed who have other drug habits as well. The main linchpin, however, is the consumption of alcohol. The majority of the people attending the scene are in their 50s or older, ethnically Danish, and about two-thirds are male. Most of them live in the immediate neighborhood; however, there are people who have a longer “commute” and occasional visitors from other parts of town or out of town who have relations to people in the scene. The number of people present changes in the course of the day and according to the time of the month (“paydays” are usually busy, the end of the month less so) and the season. As The Shed is open on two sides, the weather also plays a role. Cold, wet, and windy weather generally means fewer people, while hot, sunny days mean the opposite. The ethnographer, in the course of a three-year step-in step-out fieldwork period, noted approximately 40 named people who were spending some time in The Shed and additionally about half that number who only passed peripherally or did not want to share any information with the ethnographer.

The large majority of the people in the scene are either on unemployment benefits, early retirement pension, or pension. Almost everyone drinks alcohol; however, there are people who have “dry” patches or choose not to drink at times. Most of them buy beers or liquor at the local supermarket and take it to The Shed and drink it there. The Shed has several functions – it is both a place to spend the time and a social hub, providing the primary social network for many of the people attending the scene, but sometimes also serves as a place that offers assistance, consolation, or advice. People sometimes share beers or cigarettes, some people loan money to other people, some help others out with grocery shopping or managing finances or technical issues with e.g., mobile phones. Advice on health issues and dealing with authorities is generally shared freely. Sometimes pills or other substances change hands discreetly and a cannabis joint is shared among some of those present, in which case they often move out of The Shed to smoke.

#### 3.1.3 Disposable ties or community relations?

The local community workers and the priests working at the church often stop by The Shed to chat and to update the people in the scene on what activities are taking place and try to engage them, occasionally successfully, but most often the ones present are more comfortable staying where they are. Some of the people from the scene also occasionally go to the nearby community café for lunch and one or two have been active in communal activities like gardening and an activity group for men. For many, however, The Shed is their primary site of social interaction. As Jakob, one of the regulars, put it in an interview: “Then I drink my beers here. I don’t do much apart from going here to have a good time, and then go back in the afternoon, to look after my apartment, and that’s that”. One of the female users, Britta, put it even more bluntly:

“I sit here just as much because I have no social relations. I am a lonely person. If I wasn’t sitting here and didn’t have my pets, I would be talking to the television. I don’t have any close social relations, I have The Shed. And then I have my family, they are close relations, but they get fucking tired of me and I’m good at getting into fights with them over the phone”.

She also went cold turkey a few times during the fieldwork period, and would only pass by warily and exchange a few words with whoever was present in The Shed, when she was not drinking, but a week or two later she would be back inside The Shed. This was not because the others tried to convince her to “come back”, but at least in part because it was lonely not being part of the community.

Laurits, another steadfast regular, explained that the scene at The Shed was less volatile than other open drug and alcohol scenes in the city. “Here I can relax and drink slow and easy, instead of that race in the city, about who drinks the most and who gets drunk first, and you end up in that street life”. As such, the people attending the scene in general considered it a well-functioning open drinking scene and believed that it was known as such among professionals (police, case workers, community workers, street nurses, et cetera), which was confirmed in both formal and informal talks with some of these professionals.

The community worker also pointed to how the scene had, in her view, become an increasingly positive addition to the local community.

“They have their place here, and their community, and that’s it. And I believe it helps [to prevent conflicts], that they’re shielded. And in addition, that they seem to have decided to be accommodating and dialogue-oriented and to greet nicely when people pass by, so that their general attitude is respectful and accommodating, so that helps. And then they get much in return. And I think they are cozy”

During the COVID-19 pandemic, the people in the scene made an effort to show that they followed the recommendations from the Danish health authorities by going to be tested (there was a mobile test center nearby during part of the pandemic), by pointedly using hand-sanitizers kept in pockets, and maintaining a social distance. When the Danish health authorities banned public assemblies of more than 10 people, there was a more or less communal decision in the scene, that if more than 10 people showed up, the late arrivals would take some chairs and sit outside The Shed.

One of the regulars, Anette, a woman in her 50s, had a long history in the scene. When some of the others would try to avoid going to be tested because it was bothersome standing in line, she would prod them and scold them. She referred in an interview to how one of the old-timers, who had been a dominant figure when she was young and started participating in the scene, had instructed the others present to put their beers down, shut up, and rise to stand when there were funerals in the church and the casket was rolled into the hearse. “That is probably also why we have a good relationship with the church,” she said.

However, once in a while, internal conflicts surface among some of the people going to The Shed. The ethnographer observed and was told about several interpersonal conflicts, some of them resulting in the threat of violence or actual acts of violence, and conflicts over “proper” behavior. There were also sometimes conflicts involving people outside the scene, in a couple of cases with younger men involved in drug dealing and petty crime. Some individuals were excluded from the community of the scene and shunned by the others if they approached, if not directly threatened to stay away, because of previous violent or provocative behavior.

One example of this was referred to the ethnographer when one day one of the regulars, Jakob, was nursing a wound on his finger. When asked what had happened, Jakob told the ethnographer that a man named Mikkel had bit him “as I was throwing him out. Then I gave him a punch or two”. Anette supplemented and explained that Mikkel had been high and out of control and had been pointing a lit flashlight in the eyes of the others, and when he did not stop when the others present had asked him to, Jakob had stepped up. Everyone agreed that Jakob had been in the right and Mikkel had deserved what he got and would not show up again anytime soon. He was “quarantined” for a while and was not encountered around The Shed again.

The American sociologist, Matthew Desmond, has described how the urban poor increasingly, due to pressure on housing markets and the lack of protection for tenants, become dependent on “disposable ties” (30–32) as they are often evicted from their apartments due to behavior and being unable to pay rent on time, but also sometimes on account of landlords evicting them to make more money from other types of tenants. This has, Desmond argues, impacted poor urban communities, and the way the urban poor form relations to others, so instead of stable relationships formed over years of being neighbors, they become dependent on the assistance of people they often have only short-term relations with. While the Danish housing market has much better tenant protection, rent levels are still rising in the cities and people with low incomes and unstable living conditions are at higher risk of getting evicted. Several of the people going to The Shed had been homeless before, and a few balanced on the brink of eviction due to not being able to pay their rents, while at times one or two could be technically homeless, and sleeping on couches and in allotment huts. However, several had relatively stable living conditions and had been living in the immediate neighborhood for a long time – some of them for decades. Harald, who had lived in the neighborhood for about five years when interviewed in 2020, compared an inner-city drug and alcohol scene he had attended before moving to Kildebjerg with The Shed:

“The difference between here and [other place in the city center] is that this is my local environment, I live here. Down there you’re more anonymous and you can act up, and then go to a home somewhere else … I’d say that is a big difference. You’re not supposed to soil your own nest [DA: *skide i egen rede*]. Or there is a limit to how much you can soil your own nest.”

Laurits, usually a steadfast presence in The Shed, would, however, also go on drinking and cannabis smoking binges elsewhere occasionally. He named the people in The Shed his “friends”, whereas he mainly had “acquaintances” elsewhere, and Jakob said one the differences was that you had to be “harder” to get by in other drug and alcohol scenes; “[T]here are some people who don’t know the difference between what’s yours and what’s theirs”. The local anchoring of The Shed seemed to allow for ties that were not necessarily easily “disposable”, however volatile they could be, yet also carried the wariness that comes with the experience of having been let down and having let others down. “You have to be hard on people who drink,” Anette remarked in an interview, on the subject of giving out loans or helping people out. Harald describe the ties at the Shed in the following way: “Yes, there is care for others here, but there is no security net under that care.”. He further on pointed to the ambivalences of the alcohol and drug environment/milieu and that while people helped “hold each other up”, they also “kept each other down”. Despite the risk of being let down, however, there were several longer lasting companionships in The Shed, between people who exchanged support and formed what seemed to be strong bonds of reciprocal obligation. The Shed seemed to offer a relatively stable social space, where there was some level of general trust and where particular social codes were enforced, while its position at a local nexus also meant that behavior was adjusted somewhat to general societal codes. This will be returned to in the discussion.

### 3.2 The Bench

#### 3.2.1 How was the scene established?

For many years in a mid-sized Norwegian city in a corner of a bus station next to the railroad, two benches were located under the roof of a bicycle shelter. This is a busy area in the city center with many people passing by.

Since it has been located at the same area for more than 40 years, no one exactly remembers how it was established. It has almost been part of the city’s identity. One of the people at The Bench said that in the beginning it was occupied by people drinking alcohol, but when amphetamine came to town in the 80s, other illicit drugs such as hashish and heroin were also introduced. At that time, the scene was at the other end of the bus station. When an elderly man was rescued from a suicide attempt by one of the people at the scene, the one being rescued and the rescuer decided to make the scene more comfortable. The two of them located a bicycle shelter that no one was using at the end of the bus station, close to a parking lot. Sahlin (33) uses the term “spaces of uncertainty”, which is understood as local spaces that have no clear function, places that are almost “waiting to be used”. The two men removed the bike racks and replaced them with two benches under a bicycle shelter. Some of the people attending the scene bought flowers to decorate and make the scene look pretty. They also decided that they should keep the place tidy from littering. Since no one used the bike shed, there were no public protests. The man who was saved from the suicide attempt also created a website connected to the place now called “The Bench”, with the title “The Bench 2 die 4”. www.benken2die4.com. Now both the two men are dead, and the website has been deleted.

#### 3.2.2 From informal to more formalized scene

For many years, there has been a public discussion about what to do with the bus station and the big, ugly parking place in the middle of the city. When the municipality finely decided that the city center should be rebuilt as a park with play activities for children and families, it had consequences for The Bench long-established in this area. During the construction process, the benches were moved further away from the bus station to a drafty corner where passersby got very close to them. As a result, only a few people used the benches. The people gathering at The Bench spread, and many stayed just outside the shops at the bus station. This in turn led to complaints from the shopkeepers who believed that their customers were scared away. However, it was decided by the local community that The Bench should be included in the community rebuilding plans, with various upgrades and adaptations. The city’s mayor regularly visited The Bench to talk to those who sat there and to emphasize that in the renewal of the city center, there would also be a place for them.

At that time, our research group at The University of Stavanger informed the mayor that we wanted to write about this relocation process. We were invited to meetings with the city planners. The plan for the park was finalized, but they had forgotten to make room for The Bench. As a council member explained:

“They were a bit forgotten to begin with, but then there was input when they started building and doing things, then The Bench was moved, and then there was a lot around it, and the idea that we should then create a separate space dedicated to them came.”

When we pointed this out, a process of finding alternative locations elsewhere in the city began. Meetings were also held with people from The Bench community to get their views. They wanted to stay downtown and emphasized that if they got put somewhere outside the city center, they wouldn’t use it, as a person from the Bench explained:

“The further away they [the municipality] move The Bench [from downtown], then more drug users will return to downtown again. So, I think it will be very difficult to move it [The Bench] from the city center. If it’s far away from the city center, then they [the municipality] spread the problem across the whole city.”

None of the alternative locations were satisfactory and because of feedback from people at The Bench, it was decided to find a place in the new park at the bus station. A corner of the park was then set aside. Drawings were made of “the new bench” that were presented to the people attending the scene and minor adjustments were made, and in the spring of 2021, The New Bench was placed in the corner of the city park with a view of the new park with all its splendor (10).

#### 3.2.3 Who attends the scene?

The Bench is frequented by 70 to 80 adults, all of whom take some form of intoxicant such as hash, alcohol, various pills, and small amounts of amphetamine and heroin. Among them are only adults, with an average age of 45 years. Most of them are men – (about 75%). People who attend The Bench have been using drugs for many years. Some of them have also been former drug dealers (small dealers), but most of them are just people addicted to drugs and lowest in the drug hierarchy. Some people in the drug community call them waste users, because they use all kinds of drugs, all the drugs that are available. There is some trade in illegal drugs on the Bench, but it is very hidden. However, transactions of drugs are agreed upon there.

The persons who attend The Bench have different backgrounds. Some, such as Nina, have had a difficult childhood with neglect. Her parents had major mental health problems was a lot of substance abuse in the home.

Others, such as Ole, a man in his fifties, was well established with an education, job, and family, but started with heavy drug use later in life:

“During the recession of the 1980s, many lost their jobs, but he himself survived the recession because he worked hard. He hired himself out to a firm for assignments. It was tiring because he had to work all the time…. Then his wife took the kids and left. To cope, he started using amphetamines in the morning before going to work and then there was often some partying in the evenings. He kept going like this for about six months, then switched to harder drugs like heroin. (Field note)”

Most of the people in the scene receive disability benefits or unemployment benefits, and most of them are ethnically Norwegian men.

#### 3.2.4 Unity on the Bench

Many struggles with loneliness and mental difficulties, and for them The Bench is a place to find people to talk to and get “medication” for their difficulties. Petter came to The Bench with heavy anxiety and asked for help: “No one has pills, but they say they can get him marijuana in a hurry if he wants it. (…) He nods. (Field note)”

If you visit The Bench, you won’t see any distribution of drugs, but if you stay for a longer period, and the people get to know you, you will discover that there are some distributions of pills and cannabis, but never heroin. The main function of The Bench is to be a meeting place for people that feel they are not welcome in ordinary society. The Bench is a place where they can meet friends, where they are welcome, where they can relax and be accepted as ordinary people.

The pandemic period was hard for all people but may be harder for people in the drug community as Ole described:

“I was already alone, but then suddenly it got very much worse. Whether life is tougher, certainly. You’ve lost your entire volunteer support system. We can’t go anywhere for a visit either. We are completely left to ourselves So for me, it’s much more isolation that leads much more easily to psychosis”.

When the whole community closed, people in the drug community met at The Bench. Both charities which distributed food and healthcare personnel met them at this place. This function as a meeting place was important to avoid the feeling of being left alone.

During the coronavirus pandemic, the Norwegian population was encouraged to do its best to avoid the spread of infection. In Norway, we have a word for this: “*dugnad*” as a form of collective action where you contribute to the common good, and the people at the drug scene participated in this *dugnad*, explained by a person attending the Bench:

“Most people are concerned about it and talk about it. But I don’t think everyone is washing their hands. (…) Some don’t wash themselves as often as they should. But there are usually only a few then. Most people follow [infection guidelines], disinfect themselves.

Being addicted to illicit drugs means a hard life. People in the drug community die between 15 and 20 years earlier than most people (34). During our research at this drug scene, several people died, and we have participated in several funerals.

“Funeral. In total, there are 80 people in the church, most of them from The Bench. After the funeral, we gathered on The Bench and talked about the funeral and remembered the deceased. (Field note)”

To ordinary society, the deceased was only a drug user, an outcast. However, at The Bench, he became Anders, a person, a friend worth remembering and mourning. By grieving, one views the other as a full human worthy of the pain. Thus, it is a matter of humanization (35).

Geir, for example, had been using heroin for more than 20 years. When the ethnographer asked him why he liked to spend time at The Bench, he answered:

“It is the feeling of security and the understanding one gets from the others that makes me stay there. They understand what it is about. Many of us have experienced bad things. I, as a hardcore heroin user, and the others, we support each other, but still, we don’t trust each other completely. I trust those who are more experienced since I have known them for a long time”.

As it can be difficult to trust one another, there are some universal rules called codex (36) at The Bench, such as not stealing from each other, not reporting anyone to the police, not being stingy. Violations of these rules lead to some form of punishment, according to the seriousness of the violation. Although one can be punished for breaking rules, rules also constitute a form of security. They tie the drug community together, and people recognize each other as worthy persons. The visible presence of The Bench also means that they can look after each other if something unforeseen should happen to one of them, as one of the persons on The Bench explained: “If something happens on The Bench, there is always someone who takes responsibility, calls an ambulance or something.”

#### 3.2.5 Contact, dialogue, and integration with the local community?

Since The Bench is in the center of the city where many people are passing by, those at The Bench are visible to all who pass. People on The Bench have very close contact with the city’s inhabitants and are close to the children’s playground, something that has the potential for conflict. Still, both city hall staff and police recognized that the drug scene meets some human needs and won’t disappear on its own, and therefore they acknowledged that it exists. As one of the person from the Town hall explained:

“The fact that the municipality takes care of some of those who are lowest on the ladder, I think can actually help to change attitudes in the population, and that in a way something positive can come out of it.”

Even the police expressed an attitude toward the people on The Bench that showed an understanding for the people there, despite the closeness to the children’s park. As one of the police officers explained, it’s about not hiding people who are having a hard time:

“It’s their safe place to be, but I don’t think it has anything to do with a [ … ] children’s park [ … ]. But I think that they have to have a place to be [ … ], feel part of normal society then, in a way, I think that’s important to them.)”

The drug society knows that there are certain conditions to preserve The Bench in its current location. They have been given a place to stay, but the authorities have the power to chase them away. Therefore, they have internal rules (codex), for how to behave at The Bench. This codex was formed when The Bench was established under the bicycle shelter. One rule is that no one shoots opioids there. Arne, a person at the Bench, put it in the following way: “We can’t shoot heroin at this place, because children are passing by”. The most important rule is not to interfere with youth because it will not be tolerated by the authorities. The codex was further elaborated on by another person:

“There is a certain justice in keeping the youth away because they have no business there [at The Bench]. Don’t under any circumstances give them cigarettes, and especially not alcohol. Or other things …. They’ve [the youth] been told, by several drug users, that they should stay far away from The Bench. They have no business being there.”

It seems that the drug scene at The Bench has achieved a good combination of internal justice and relation to the environment. In a way, they’re their own police. They sort things out internally so that the police don’t have to correct them. They have a sort of common understanding with the police of how to behave. As a police officer explained:

“We have a focus on presence, having a dialogue with users. It’s important that we kind of manage to create such a common understanding of how we want it, and that they kind of have the same understanding. That’s kind of what we had before.”

People at The Bench want to be a part of society. The closeness to society increases the interaction between the people at The Bench and society, which has the potential to reduce stigma, expressed in the following quote by a person spending time at the Bench:

“When we have been allowed to sit on The Bench in peace, so close to the cityscape, and people see that even if we are here, we’re not dangerous … I see the interaction between the drug community and ordinary people has improved.

### 3.3 Commonalities and differences between cases in Denmark and Norway

The two sites have several resemblances; however, they also differ in some respects. Both are centered around a shed-like structure, offering some shelter from rain and wind, and providing the people who spend time there with a place to sit. One interesting characteristic of both scenes is that they have gone through a process from being informal scenes to more formalized scenes over the past decades. These processes have happened through dialogue and negotiations with the local community and are, in this respect, accepted and not subject to imminent police interventions or dispersion. Still, the level of formality is different; the Norwegian Bench has been through local political and administrative practices on municipal level, while the Danish Shed has primarily been formalized hyper-locally, with the lot owners (housing associations, shop owners, and local church) around the suburban square as the driving force. That is also mirrored in the aesthetics of The Shed and materials of the structure – which is all about functionality, whereas the new Bench has been “designed” by architects as part of a city upgrade. Both scenes have been acknowledged as a safe place for the people attending, where they are allowed to be a part of society.

Both scenes are primarily local, but whereas the Norwegian Bench attracts people from the small city and nearby towns, the Danish Shed attracts primarily people from the suburb where it is situated and neighboring suburbs and a few non-locals with connections to locals from the scene. The number of persons (approximately around 80) gathering at the Norwegian Bench over a period of time are somewhat higher compared to the number (approximately 40) gathering at the Danish Shed, and they are slightly younger. Most of them are men (75%), and there are fewer women. Both scenes can be considered as relatively small, with seldom more than 10-15 people gathering at the same time, and often fewer.

While drugs have previously played a larger part in the Danish scene at The Shed, it is now primarily centered around the use of alcohol, predominantly beers (both regular and strong beers), but also strong liquor like vodka and whisky and to a lesser degree marihuana and pills (opioids, benzodiazepines) and harder drugs. Some of the people from The Shed use harder drugs, but rarely at The Shed. Most people gathering at The Bench are polydrug (marginalized drug) users, and maybe half of the people are part of a methadone program and strive to cover their “side-use” of other drugs. Still, likewise as at The Shed, the social interactions at The Bench are centered around drinking alcohol and smoking an occasional joint. No direct drug dealing characterizes the scenes; however, “social dealing” (37) is a phenomenon at both sites, where people share or distribute smaller amounts of drugs (mainly weed and prescription drugs) to people they know. Most people gathering at both sites have a permanent residence. Still, some of the people have a more temporary situation, living with different friends or at a shelters and temporary housing.

It is also interesting to note that both scenes have existed for a long time and have an aging population (people in their 40s and 50s), and neither of the scenes seems to attract or recruit young people. In both scenes, there seems to be a focus on keeping people who are considered too young away from the scene, while the scenes might also simply not be particularly attractive to young people, as they consist of mainly older adults and offer limited facilities. Most of the time, it is just people sitting and talking and drinking. In addition, other locals take notice of who hangs out at the scenes and there is some level of stigma attached to these places.

Both scenes have connections to social and local community services. In Denmark, there are contact between the scene and local community workers, and people from the local church dropping by, but also at times with municipal outreach workers, while the major organizations working with marginalized groups seem to concentrate their efforts in more central drug and alcohol scenes in the city. The Norwegian scene is more closely attached to outreach services and different kinds of low-threshold support.

## 4 Discussion

### 4.1 Characteristics of smaller open alcohol and drug scenes: a different phenomenon?

Compared to the common definition of an open drug scene as a situation where citizens are publicly confronted with drug use and drug dealing (2, 3), both the Norwegian Bench and the Danish Shed are not mainly defined by these kind of practices. The main substance consumed at the scenes is alcohol, not illegal drugs. The scenes are not designated places for drug dealing, although social dealing is part of what goes on. The main purpose of attending the scene is not to get hold of drugs but something else; for quite a few people, the scene is a primary site of social interaction, and the scene is all they have. So, are we looking at a different phenomenon than that established as an open drug scene? The scenes described in this study have existed for decades, and the persons frequenting them are mainly in their 40s and 50s. While these factors seem to make the scenes more socially stable, they also mean that, as obvious in the Norwegian example, a large proportion of the people die or become physically impaired. There seemed to be little expectation of future improvements in life situations for the people of both scenes, and while the scenes had become less chaotic, it did not necessarily mean that the people going there had become less marginalized over the years. What does it mean that the people attending the scene are growing older? Has distribution of drugs gone elsewhere?

In Bless et al. (2) and the EMCDDA (4) study, all of the cities studied considered the open drug scene as a problem to be managed. Magnusson (5), in researching open drug scenes in the Swedish context, has even integrated this element into her definition of an open drug scene: “a geographic area, sustained in space and time, where use and dealing of drugs takes place in the public and is perceived as problematic by authorities and/or the public” (5, p. 306). Maybe this is an interesting difference regarding the cases in this study. Both scenes have traditionally, back in time, been considered a problem by the public regarding nuisance, legality, and push and pull factors (e.g., recruitment to drug careers). In the Danish case, the people who now go to The Shed used to hang out in front of the local supermarket and church, and The Shed was constructed to make them less of a “nuisance” to other people from the neighborhood who were going to church, grocery shopping, or delivering or picking up their kids at the nearby kindergartens. Thus, through dialogue with the local community, an understanding has been developed, so that the scene is accepted in a mutual understanding of rules, codex, and borders. Similar processes have been occuring at the Bench. In a way, you can say that these scenes have evolved and have been “solved” by being defined as accepted places to be, due to the people obeying certain nonwritten rules.

### 4.2 Spaces of social connectedness, decency, and ambivalence

#### 4.2.1 A place of performing decency?

Another interesting aspect of these smaller open alcohol and drug scenes is how the public nature of the locations where the scenes are centered, combined with the fact that they are part of smaller city center where people recognize each other, might influence how the people present at the scenes behave. Both at The Bench and at The Shed, there were unwritten rules about how to behave, not only related to internal issues in the group, but also about general behavior at the location in relation to the local community. Goffman (38) famously described how people perform and express themselves differently depending on whether they are “backstage” or “frontstage”; people can play different roles depending on the social context. Understanding The Shed and The Bench respectively as both frontstage and backstage simultaneously might help us understand some of the ambivalences of the scenes.

As previously mentioned, the interviewee Harald told the Danish ethnographer: “You do know they’re on their best behavior when you’re there, right?”, meaning that the presence of an outsider in The Shed changed the way people spoke to each other and behaved. The Shed shifted from backstage to frontstage, a place of performance of particular behavior. It took many hours of fieldwork before the Danish ethnographer started observing the use of cannabis and sharing of illegal substances, and was told stories about violence, theft, and internal conflicts. The regulars at The Shed always took care to greet people passing by, make joking remarks, and pointedly sweep and clean out the shed occasionally. During the coronavirus pandemic, social distancing was performed and the people at The Shed made sure to stand in line and get tested at the local test center. This was in part because they feared contracting the virus, but also because, as Anette said, they wanted to show that they were responsible citizens and not careless.

The performance of decency was reinforced in the Norwegian case in the dialogue and contact with local politicians. It was an interesting process when the scene was accepted, and it did something for the people attending the scene. The Norwegian researchers noted a form of inner justice – you do the “police” work internally. The “contract” with the local community, related to the “formalization” of the scene, is based on a certain trust that things will not get out of hand. For example, people kept the place tidy, throwing empty cans of beer in the bin or returning them for packaging deposits. Like in the Danish case, the people at The Bench made a point of being tested and following coronavirus regulations, doing their share of collective action.

#### 4.2.2 A place of social connectedness

American sociologist Mattew Desmond (30) points to a development in sociological literature about the American urban poor; from the end of the 19^th^ century up to the 1990s, with Carol Stack’s *All Our Kin* (39) as a prime example, American poor inner-city neighborhoods were described as having strong domestic ties built on an ethics of reciprocity and mutual obligation among their populations (30, p. 1297). However, from the 1990s and onwards, Desmond identifies a number of studies that point to increased distrust in poor neighborhoods and a “mounting evidence against the saliency of kin” (30, p. 1298). Instead, Desmond identifies what he calls “disposable ties” (30, p. 1299) employed by poor families to replace kinship ties, and explores how these ties are formed, used, and often discarded.

The two open alcohol and drug scenes in Norway and Denmark both function as spaces of social connectedness, offering some form of security and support for the people who go there. As an example, during the pandemic, The Bench was one of the few places that people actually gathered and supported each other. To what extent are the ties between the people of the scenes to be understood as “disposable”? As pointed out in the earlier section on The Shed, these scenes seem to create steadier, yet still fragile, volatile, and vulnerable ties based on kinship, romantic relationships, and companionships.

As referred earlier: Harald pointed to the ambivalences of the alcohol and drug environment/milieu and that while people helped “hold each other up”, they also “kept each other down”. Similarly, at The Bench, Geir articulated how they support each other at the same time as they don’t trust each other completely. The rituals and the connectedness at the scene could draw people in (7), but it also made it difficult to leave. And we have also seen how Britta when she tried to sober up, still came back, in part because it was lonely not being part of the community.

While it was possible to find community and care in The Shed and at The Bench, the community was not always able to be supportive when people searched for ways out of drinking and using drugs, and the code of a tight knit community can also mean that some people get expelled, like in the example from The Shed. Care cannot be counted on either – as Britta from The Shed experienced when she broke her foot and needed help grocery shopping and having her dog walked. That did not come for free; she had to pay people to do it. There is usually an expectation of something in return for favors offered, even if it is only sharing cigarettes and beer with people who are out of money; this may only not be the case between close companions.

#### 4.2.3 Stigmatized spaces – destigmatizing processes?

Both the Danish Shed and the Norwegian Bench can be seen as stigmatized spaces, as it makes people and their problems very visible, but also de-stigmatizing, as they give people a place of their own and some acceptance and recognition of their existence. They have a place in society – but still it is also outside society, or liminal.

There is an ambivalence to sitting in The Shed or on The Bench. One is visible, stigmatized – but still gaining social connectedness. At The Bench, a change was experienced regarding stigma when someone with authority (the mayor) went public and announced that the city was for everybody, including people on The Bench. The mayor was proud that they had provided a place – the new Bench – for those less fortunate. Both people at The Shed and on The Bench know that they are seen – by the public and by the police – and in this way have to follow the rules and behave. At The Shed, the acceptance from community workers, police, and local residents meant that the people there felt somewhat accepted, but they were also very well aware that people took notice of who was hanging out there.

Both at The Shed and at The Bench, local community workers, outreach workers, and people from the church often pass by. They might tell people at the scenes about activities or what is on the menu at the local social café, or ask about people they are worried about, or cases they need to follow up. The people from The Shed are allowed to use the bathrooms at the church and the local community center, making it much less of a hassle for particularly the women to go to the toilet. While the people from The Shed rarely accept the offers to attend activities, occasionally some go to the social café for meals, and one or two began migrating to other activities during the fieldwork period and managed to cut down on drinking and using cannabis. People at The Bench are regular users of low-threshold services in the city. While there are organizations offering treatment and support for marginalized substance users, it can still be difficult to distance oneself from a particular community, like the one at the Bench or at the Shed. This is not to discount the work being done by user organizations and other actors. However, while some of the users of the Shed occasionally made use of low-threshold facilities in other parts of the city, they often did so for short stretches. For many of the users of these sites, the alternative to the Shed or the Bench often seemed to be a form of self-administered abstinence and self-isolation.

The studies show the potential that lies in performing dialogue and engaging in co-creation processes between different groups of people in society. Such processes contribute to reducing the stigma attached to marginalized groups. In the two cases, these processes have taken place at different levels of society, but how the involvement of people in power, such as the mayor, makes the dialogue even more significant by reaching the attention of a wider group of people.

## 5 Conclusion

In this article, we have taken the first steps in a comparative analysis between two open alcohol and drug scenes – The Shed in Denmark and The Bench in Norway. As we have attempted to show, there are several similarities between the two scenes in terms of their history, their size, and the development of the scenes over time. The scenes are relatively small, and while the Norwegian Bench has a central location in a smaller city, the Danish Shed has a central location in a suburban neighborhood of a city. We argue that these more intimate scenes allow for the building of stronger relational bonds between the people going there. We further argue that the people of these scenes seem to be more concerned with behaving with some level of decency because they have come to be somewhat accepted by local society and have a place they do not wish to lose. There seems to be a reduction of perceived stigma in this for the people of the scenes. They do not feel as exposed as in other public locations.

This does not mean that there is no stigma attached to sitting at The Bench or in The Shed. The people of the scenes are aware of this and are ambivalent about it. While the scenes offer community and care, conflicts also unfold, and people can be expelled and shunned if they step out of line.

This study contributes to the research literature by highlighting the aspect of social connectedness and meaning making for the people involved in open alcohol and other drug scenes. It further contributes by expanding the understanding of the phenomenon of open alcohol and drug scenes in urban environments. Smaller alcohol and other drug scenes are somewhat different from an “open drug scene” and share other characteristics and other functions for the people involved.

The similarities between the two scenes described here point to the potentials of the comparative angle when working with qualitative data gathered through extensive fieldwork and qualitative interviews. While we have taken important first steps here, we believe there is still much potential in comparative research to do in a Nordic context, focusing on 1. the prevalence, characteristics, functions and meanings of smaller alcohol and drug scenes, 2. the differences, similarities and the development or closure of respectively smaller and bigger scenes in the Nordic countries.

The study further highlights the importance of dialogue and communication between people attending these scenes and the wider community, using the potential for a reduction in the public stigma related to these spaces and the people attending them.

### 5.1 Limitations

The two cases compared here are particular in each their own way, and data collection was not initially prepared or organized with a comparative framework in mind. The comparative analysis is thus based on existing data sets that cover different time periods and two different locations in two different countries. However, we believe the comparative approach has offered new and interesting insights across the two cases as examples of how smaller open alcohol and drug scenes function.

## Data availability statement

The data analyzed in this study is subject to the following licenses/restrictions: the datasets was collected by the researchers in this study, and are restricted to be used by them after consent from the participants. Requests to access these datasets should be directed to JSB, jsb.crf@psy.au.dk and TEG, trond.gronnestad@uis.no.

## Ethics statement

The fieldworks were conducted in accordance with the Norwegian and Danish standards for privacy regulations and research ethics. All persons present at the scenes were informed about the researchers' presence and the research projects. Data collection using participant observation relied on field notes, not audio recordings. People being interviewed provided a written consent of their participation in the research, following the Norwegian and Danish standards for safeguarding privacy regulations and research ethics.

## Author contributions

JSB: Writing – original draft, Writing – review & editing, Data curation, Conceptualization, Investigation. TEG: Writing – original draft, Writing – review & editing, Data curation, Conceptualization, Investigation. ASS: Writing – original draft, Writing – review & editing, Data curation, Conceptualization, Investigation. VHB: Writing – review & editing, Data curation. AS: Writing – review & editing, Data curation.
